# EpiBrainRad: an epidemiologic study of the neurotoxicity induced by radiotherapy in high grade glioma patients

**DOI:** 10.1186/s12883-015-0519-6

**Published:** 2015-12-18

**Authors:** Thomas Durand, Sophie Jacob, Laura Lebouil, Hassen Douzane, Philippe Lestaevel, Amithys Rahimian, Dimitri Psimaras, Loïc Feuvret, Delphine Leclercq, Bruno Brochet, Radia Tamarat, Fabien Milliat, Marc Benderitter, Nicolas Vayatis, Georges Noël, Khê Hoang-Xuan, Jean-Yves Delattre, Damien Ricard, Marie-Odile Bernier

**Affiliations:** UMR CNRS 8257 SSA MD4 Cognition and Action Group, 45 rue des Saints Pères, 75270 Paris CEDEX 06, France; Service de neurologie Mazarin, hôpital de la Pitié-Salpêtrière, 47-83 boulevard de l’Hôpital, 75013 Paris, France; Institut de Radioprotection et de Sûreté Nucléaire (IRSN), PRP-HOM, SRBE, 31 avenue de la Division Leclerc, 92260 Fontenay aux Roses, France; Institut du Cerveau et de la Moelle, 47-83 boulevard de l’Hôpital, 75013 Paris, France; Unité de neuroradiologie diagnostique et fonctionnelle, hôpital de la Pitié-Salpêtrière, 47-83 boulevard de l’Hôpital, 75013 Paris, France; Service de Neurologie, groupe hôspitalier Pellegrin, place Amélie Raba-Léon, 33076 Bordeaux, France; UMR CNRS 8536 Centre de mathématiques et de leurs applications, ENS Cachan, 61 avenue du président Wilson, 94235 Cachan CEDEX, France; Département de radiothérapie, centre de lutte contre le cancer Paul Strauss, 3 rue de la porte de l’hôpital, 67065 Strasbourg CEDEX, France; Service de neurologie, hôpital d’instruction des armées du Val-de-Grace, 71 boulevard de Port-Royal, 75005 Paris, France

**Keywords:** Radiotherapy, Cognitive impairments, Neurotoxicity, Leukoencephalopathy, Quality-of-life

## Abstract

**Background:**

Radiotherapy is one of the most important treatments of primary and metastatic brain tumors. Unfortunately, it can involve moderate to severe complications among which leukoencephalopathy is very frequent and implies cognitive deficits such as memory, attention and executive dysfunctions. However, the incidence of this complication is not well established and the risk factors and process are poorly understood. The main objective of the study is to improve knowledge on radio-induced leukoencephalopathy based on pluridisciplinar approaches combining cognitive, biologic, imagery and dosimetric investigations.

**Method/Design:**

The EpiBrainRad study is a prospective cohort study including newly diagnosed high grade gliomas patients treated by radiotherapy and concomitant-adjuvant temozolomide chemotherapy. Patients are included between their surgery and first day of radio-chemotherapy, and the follow-up lasts for 3 years after treatment. Cognitive functioning assessments, specific blood biomarkers measures and magnetic resonance imagery are performed at different moment during the follow-up, and a specific dosimetric assessment of organs involved in the beam fields is performed. Firstly, leukoencephalopathy incidence rate will be estimated in this population. Secondly, correlations between cognitive impairments and dosimetry, biomarkers ranges and anomalies on imagery will be analyzed in order to better understand the onset and evolution of cognitive decrement associated with radiotherapy. Furthermore, a new cognitive test, quickly and easily performed, will be studied to determine its sensibility to detect leukoencephalopathy decrement.

**Discussion:**

With an original multidisciplinary approach, the EpiBrainRad study aims to improve knowledge on radio-induced leukoencephalopathy in order to improve its early diagnosis and prevention. The main challenge is to preserve quality-of-life after cancer treatments which imply to study the incidence of radiation-induced complications and their associated risk factors.

**Trial Registration:**

NCT02544178

## Background

Radiotherapy is one of the most important treatments of metastatic and primary brain tumors of which 60 % are high grade glioblastomas. With the recent development of modern irradiation techniques, survival of patients has increased and mid- to long-term side effects became more visible, such as neurologic complications [1]. Among these complications, leukoencephalopathy seems to be the most frequent. It is characterized by a progressive and diffuse demyelination, an axonal loss and vascular lesions [2]. Consequences of leukoencephalopathy involve cognitive deficits which dramatically reduce the patient’s quality-of-life [3–5]. Thus, since the last few years, the neuropsychological status represents an important issue in clinical trials as well as in individual outcomes [6]. Long-term memory, information speed processing, attention and executive functions are recognized to be the most sensitive functions to be affected by radiations [2, 7–10]. However, there is no consensus to describe the main evolution of cognitive decrement following radiotherapy because of important differences in studies’ protocols (assessment time, decrement definitions, material). The radiation-induced leukoencephalopathy incidence is thus difficult to estimate precisely and varies from 30 to 50 % [10, 11] according to the length of follow-up. Moreover, available studies are often retrospective which may induce bias and based on small samples which limits statistical robustness of results.

Although several risk factors of neurotoxic complication have been identified such as patient’s age, tumor location, total dose of radiation, fractionation, field size [2, 9, 12], the pathophysiology of radiation-induced neurotoxicity is still poorly understood. It could involve inflammation, blood barrier disruption, vascular lesion, demyelination, radio-necrosis and edema [2, 9]. Until now, it is impossible to precisely estimate at individual level the risk for a patient to develop this neurotoxic complication [13, 14]. Some individual risk factor such as cardio-vascular diseases (hypertension, diabetes), smoking, old age [15, 16] seem implicated in leukoencephalopathy. Assessment of the impact of both individual risk factors and treatment toxicity requires to study a large group of patient with prospective collection of accurate data and follow-up.

From a biological point of view, biological mechanisms of the initiation and progression of cognitive impairements is not well known. However, specific biomarkers predictive of the cognitive impairments in this population would help in screening patients at risk of neurotoxicity. Currently, biomarkers of neurotoxicity have been poorly studied. Thus, several markers deserve to be explored such as the S-100B protein known to be a marker of traumatic cerebral lesion, neurodegenerative diseases and aggressive gliomas [17], specific isoprostanes (as 8,12-*iso*-iPF_2α_-VI) implicated in the oxidative/nitrate stress in neurodegenerative diseases [18] and homocystein which plasma concentration has been associated with poor memory performances in old individuals [19]. Furthermore, new biomarkers seems to be potentially very interesting. Indeed, micro RNA and microparticules have been identified as potential biomarkers of neurological defects [20, 21]. Then the strategy proposed is to test classical and new biomarkers in order to individualize those, which could be accurate for the screening of patients at risk of cognitive impairments.

At last, assessment of cognitive impairment is difficult because the exploration of cognitive side effects is not systematic and often depends on patients’ complaints and clinicians’ sensibility to this aspect. A formal neuropsychological assessment can’t be performed in routine, as often as it should be for an adapted follow-up of brain tumor patients. Indeed, it is time consuming, needs expert professionals and validated material, and consequently it is not easily implemented in most medical center. A new tool for exploration of cognitive deficits is the *Computerized Speed Cognitive Test* (Legal deposit: IDDN.FR.001.180018.000.S.P.2014.000.31230) (CSCT) [22]. The CSCT was first developed to assess information speed processing in multiple sclerosis. Because it presents a low learning-effect and focuses on a cognitive characteristic often impaired in neuro-oncologic population, it could be used to detect a cognitive deterioration during repeated medical visits, and thus be used as an alarm to start a more extensive formal assessment. Then, it is suitable for the follow-up of a cohort of patients treated for brain tumor and allows to give quick and reliable information on cognitive impairment, compatible with the usual clinical follow-up of the patients.

### Objectives

The purpose of the EpiBrainRad study is to establish a cohort of patients treated with radiotherapy for a high grade glioma.

The main objective is to estimate the cognitive impairment incidence related to radio-induced leukoencephalopathy in this population.

Secondary objectives are:

- To study the impact of associated risk factors on leukoencephalopathy development, including individual factors and treatment.
- Biologic markers of neurologic degradation will be studied to evaluate the correlations between cognitive impairments and biological abnormalities.
- Specific organs dosimetry (such as hippocampus, temporal lobes, corpus callosum, peri-ventricular white matter, posterior fossa) correlated with radiologic abnormalities on magnetic resonance imagery (MRI) and clinical symptoms will be analyzed to better understand the evolution of radio-induced leukoencephalopathy.
- Finally, the assessment of the sensibility and specificity of a quick cognitive test, *the Computerised Speed Cognitive Test* [22], to detect a cognitive decrement during the follow up of the patients.

## Methods/Design

### Study design and patient selection

The EpiBrainRad study is an observational prospective cohort. Patients’ recruitment takes place at *Hôpital de la Pitié-Salpêtrière (Paris, France)* and *Centre de Lutte Contre le Cancer Paul Strauss (Strasbourg, France)* for 2 years and each patient is followed during 3 years. The cohort includes newly-diagnosed adults treated for a high grade glioma according to the procedure described by Stupp et al. [23]. According to patient’s age, the radiotherapy regiment can be modified to 40 gy in 15 fractions to ensure a better tolerance in elders [24]. Inclusion and exclusion criteria are listed in Table 1. Data are prospectively including clinical features, MRI images and results, blood samples, dosimetry and cognitive assessments during the usual medical follow-up.

**Table 1:** Inclusion and exclusion criteria

Inclusion criteria	Exclusion criteria
• Newly diagnosed grade III or IV glioma • Age ≥ 18 • Treated by radiotherapy and concomitant-adjuvant Tomozolomide as described by Stupp et al. [23] or with an abbreviated course of radiotherapy in elders patients [24] • Treatment and clinical follow-up performed at *Hôpital de la Pitié-Salpêtrière (Paris, France)* or *Centre de lute contre le cancer Paul Strauss (Strasbourg, France)*	• History of other CNS tumor • History of neurologic and/or psychiatric disease involving cognitive impairments (TBI, Stroke, mood and personality disorders…) • Insufficient understanding of French language

### Ethical approval

This study has received ethical approval from the *Comité de Protection de la Personne de Paris VI Ile de France* in December 2014 (ID: CPP/132-14) and from National Agency for Medical and Health products Safety (Reference: 2014-A01697-40). The Clinical Trial Registration Information is available at http://www.clinicaltrials.gov (Unique identifier NCT02544178). Participants enrolled in the study provide their written informed consent.

### Study endpoints

The primary endpoint is the reduction in performance on cognitive tests defined as follows: After exclusion of local recurrence, decreased detailed neuropsychological assessment score compared to the patient's baseline score before radiotherapy for at least 2 cognitive domains.

Secondary endpoints are:

- Differences in biomarkers’ concentration between the measurements before RT and: at the end of the RT, RT + 4 weeks, RT + 12 months and RT + 36 months.
- Changes in MRI images compared to the baseline imaging.
- Correlations between dosimetry, cognitive decay and MRI abnormalities onset after RT.

### Procedures

**Fig. 1:**
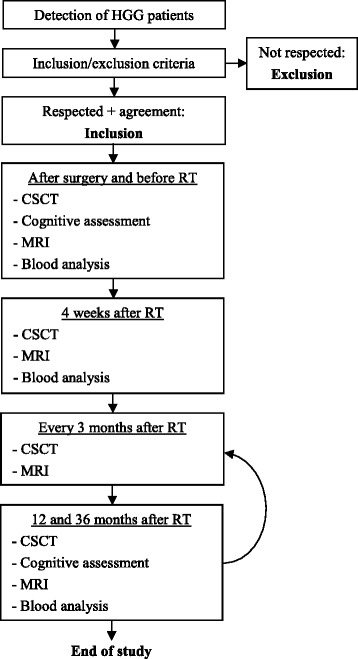
Study procedure

Figure 1 gives an overview of the study flowchart. Patients’ inclusion occurs after surgery and before radio/chemotherapy (Baseline). At this time, individual information including demographic information, medical background and tumor characteristic are collected.

Every 3 months, once the MRI of the patient is done, a medical visit occurs during which a short cognitive assessment is performed (the CSCT).

Cognitive tests, scales and questionnaires used for the formal assessment are listed in Table 2. An extensive evaluation lasting 2 h is performed with a trained neuropsychologist, followed by self-reported and caregiver-reported scales and questionnaires. The explored domains are intellectual efficiency, general functioning, verbal and visual episodic memory, attention, executive functioning, language, visuospatial and visuoconstructive abilities, mood, fatigue, quality-of-live, subjective complaints and autonomy. The formal assessment occurs at baseline and yearly after radiotherapy. In case of CSCT score decrement during follow-up the next yearly formal assessment is performed earlier. CSCT decrement is defined as a loss of 1 standard deviation compared to baseline (given its low learning-effect and good stability in controls [22]), during two consecutive assessments. The necessity of detecting a decrement during two visits permits to insure that a variation in score is not due to a transitory cause like an infection or fatigue, which is reversible and not linked to leukoencephalopathy. This procedure will allow to study the nature, course and incidence of cognitive decrement following radiotherapy in this population. At baseline and during the follow-up, the CSCT will be compared to the formal cognitive assessment to study its sensibility to detect impairments in this context.

**Table 2:** Tests and questionnaires used for the cognitive, mood and behavioral assessment

Domain	Tests, scales and questionnaires
Cognitive assessment	
Intellectual efficiency	National Adult Reading Test (French version) [38]
General functioning	Mini Mental State Examination [39]
	Montreal Cognitive Assessment [40]
	Dementia Rating Scale-2 [41]
Verbal episodic memory	RLRI-16 (Adaptation of the Grober-Buschke test) [42]
Visual episodic memory	Rey-Osterrieth Complex Figure [43]
Executive functions and Attention	Digit Span forward [44]
	Digit Span backward [44]
	Letter-number sequencing [44]
	Categorical and phonetic word fluency [45, 46]
	Trail Making Test [45, 46]
	Computerized Speed Cognitive Test [22]
	Stroop color-word Test [45, 46]
Langage	Boston Naming Test [47]
	Token Test [48]
Visuospatial and visuoconstructive functions	Rey-Osterrieth Complex Figure [43]
Self-reported Scales	
Mood	Anxiety questionnaire of Goldberg [49]
	Center for Epidemiologic Study Depression Scale [50]
Fatigue	FACIT Fatigue Scale [51]
Memory complaint	MacNair and Khan questionnaire [52, 53]
Quality-of-life	EORTC QLQ-C30 [54]
	EORTC QLQ-BN20 [55]
Care-giver-reported Scales	
Dysexecutive syndrome	ISDC [45, 46]
Autonomy	EIADL [56]

Baseline and trimensual MRI during the medical follow-up are used for extra analysis in order to study white matter abnormalities and cortical atrophy. Moreover, tumor response or progression will be assessed by the Response Assessment in Radio-Oncology working group (RANO) Criteria [25]. The white matter lesion quantification will follow the procedure described by Wahlund et al. [26] using a 4 points scale (0: no lesion; 1: focal lesions; 2: beginning confluence of lesion; 3: diffuse involvement of the entire region). The cortical atrophy quantification will follow the procedure described by Pasquier et al. [27] using a 4 points scale (0: absence of atrophy; 1: mild atrophy; 2: moderate atrophy; 3: severe atrophy). White matter lesion and cortical atrophy is assessed for all key region listed in Table 3. A double blind lecture of the MRI will be done in order to quantify these anomalies. Radiological outcomes will then be correlated with cognitive outcomes.

**Table 3:** Regions prospectively explored on MRI and dosimetric analysis

Key regions investigated		
	MRI	Dosimetry
Frontal lobes	*	*
Temporal lobes	*	*
Parietal lobes	*	
Occipital lobes	*	
Cerebellum	*	*
Basal ganglia	*	
Brain Stem	*	*
Hippocampus		*
Corpus Callosum		*
Periventricular white matter		*

* anatomical region investigated

The need for a biological marker of cognitive disorders has proven to be urgent, both for diagnosis and for monitoring of the diseases. Among the advantages that such markers may bring, we emphasize the possibility of early and even preclinical diagnosis of the disease with the subsequent correct treatment of disease by the medical team. Biomarkers for cognitive impairment have been an issue in recent years. The value of a variety of these markers, like amyloid beta or tau, has been evaluated and discussed [28]. Besides proteins, microRNAs have also demonstrated their potential as non-invasive biomarkers from blood and serum for a wide variety of human pathologies [29]. A deregulation of microRNA expression might be involved in neurological dysfunction or neurodegenerative processes. A recent serum profiling of Alzheimer disease patients provided evidence that expression changes of circulating miRNAs may be valuable biomarkers for this neurological disease [20]. In addition, microparticles have been implicated to have pathological roles in many diseases such as Alzheimer’s disease. The consensus among recent studies is that increased levels of specific types of microparticles in plasma may represent reliable biological markers for the onset and progression of central nervous system diseases [30].

Consequently, plasmatic classical biomarkers as S-100B protein, isoprostane 8,12-*iso*-iPF_2α_-VI, homocysteine and new ones, as micro RNA and microparticules (see Table 4) will be sampled in blood at baseline, 4 weeks, 12 months and 36 months after the end of radiotherapy. These biomarkers could be potential blood predictors of cognitive impairments [17, 19–21] and thus their concentration evolution after radiations will be analyzed. Additional blood sample tubes are stored to constitute a biological collection for future investigations about others biomarkers.

**Table 4:** Biomarkers sampled in blood

Classical biomarkers correlated with cognitive impairments	S-100B protein [GenBank : NP_006263]
	8,12-iso-iPF2a-VI isoprostane
	Homocysteine [GenBank : NP_060084]
	microRNAs
New biomarkers	brain-miR-112 [GenBank : AF480510]
	brain-miR-161 [GenBank : AJ535829]
	hsa-let-7d-3p [GenBank : LM380164]
	hsa-miR-5010-3p [GenBank : LM38284]
	hsa-miR-26a-5p [GenBank : LM378769]
	hsamiR-1285-5p [GenBank : LM383022]
	hsa-miR-151a-3p [GenBank : LM379262]
	hsamiR-103a-3p [GenBank : LM378788]
	hsa-miR-107 [GenBank : LM378791]
	hsa-miR-532-5p [GenBank : LM379811]
	hsa-miR-26b-5p [GenBank : LM378770]
	hsa-let-7f-5p [GenBank : LM378754]
	microparticles

Finally, histograms of dose-volumes (HDV) of specific organs and areas are collected to study the relation between the radiation doses received by these structures and the cognitive decrements observed. Indeed, some authors showed correlations between specific structures and cognitive performances [31, 32] but few data are available and thus these relations need to be explored. More, dosimetry is compared with onset of abnormalities on MRI to improve knowledge about radio-induced leukoencephalopathy process. Structures prospectively investigated are listed in Table 3.

### Statistical analysis

#### Sample size consideration

The main objective being to estimate the incidence of occurrence of neurological complications in the cohort, with an expected proportion P varying from 0.2 to 0.5, for a confidence level of 95 %, with alpha risk of 5 %, the number of subjects to be included varies between 246 and 384. Given the risk of relatively early death related to the disease, the inclusion of 400 patients will provide good statistical power for this purpose.

#### Planned analysis

The combination of data from four axes (Clinical Epidemiology, Dosimetry, Biomarkers and Radiobiology) will give the opportunity to precisely analyze the links between cognitive impairments and changes in biomarkers depending on the precise dose absorbed at different brain areas during radiotherapy brain cancer.

Descriptive statistics (means, standard deviations, percentages and 95 % confidence intervals) will be used to describe the distribution of subjects according to each of the terms of the variables studied. Analyses of variance to one or more classification criteria will be used to compare the means of continuous quantitative variables. Chi-square test or Fisher exact test will be used to compare the distribution of nominal discrete variables and / or ordinal. All tests will be bilateral with alpha = 5 %. Details of the analyzes are shown below:

#### Description of the population at baseline (before radiotherapy)

This description will include:

- Medical data collected in the questionnaire, including in particular the baseline risk factors of neurological complications.
- All biomarkers
- The MRI abnormalities
- The results of CSCT and complete cognitive assessments.

The results will be presented for the complete sample.

#### Dose assessment

This reconstruction will be made for each patient based on data from radiotherapy treatment plan. Histograms of dose/volumes will be retrieved for each structures of interest listed in Table 3 in order to study correlations between anatomic regions and neuropsychological dysfunctions.

#### Analysis of evaluation criteria

Based on primary endpoint definition, the estimate of the incidence of cognitive impairment related to radio-induced leukoencephalopathy in our cohort will be made.

An analysis of the dose–response relationship between radiation dose absorbed by different brain structures and cognitive abnormalities observed after radiotherapy will be made using a multivariate Cox model taking into account the delivered dose to the organ of interest and the time occurrence of cognitive complications. An adjustment on risk factors for cognitive complications will be achieved. Similarly for patients with cognitive impairment before radiotherapy, an analysis of potential associated risk factors will be carried out with the use of a multivariate model.

Secondary judgments criteria, including changes in MRI images relative to the reference imaging and changes in series of biological markers between measurements before RT, 4 weeks, 12 month and 36 months after RT, will be analyzed by parametric distribution comparison tests (Z) or nonparametric if needed (Wilcoxon tests).

In addition, an analysis will be conducted of the sensitivity and specificity of the CSCT to demonstrate cognitive impairment by comparing the CSCT performed before radiotherapy and detailed neuropsychological assessment (gold standard) achieved at the same time. More, CSCT decay during follow-up will be analyzed by comparing its results to formal assessment during years after treatment, to study its sensibility in detecting cognitive decrement after radiotherapy.

### Time plan

Initial inclusion data collection began in April 2015 and will continue through 2017. First statistical analysis about cognitive impairment related to radio-induced leukoencephalopathy incidence will be available in the end of 2016.

## Discussion

This study should improve our knowledge on neurologic complications of radiotherapy through an original multidisciplinar approach combining cognitive, biologic, imagery and dosimetric investigations. Our primary objective is to precisely explore cognitive impairments present before radiotherapy and to follow their worsening, or the onset of new impairments not present at baseline, during the first years after the treatment. The collection of data from different nature could be very useful to understand the leukoencephalopathy process and risk factors associated. Indeed, the assessment of cognitive defect linked to leukoencephalopathy could be obscured by the progression of the disease. Then, precise assessment of the type of cognitive defect associated with information on dose received by organs of interest and MRI results will be a major issue to study the dose response relationship between leukoencephalopathy development and radiation exposure to the brain.

Chemotherapy is known to induce cognitive impairments as well and produce a synergistic effect when administrated in combination with RT [2, 12]. Unfortunately, the treatment protocol received by our patient population doesn’t allow to specifically study the participation of chemotherapy in cognitive decrement. Nevertheless, chemotherapy related impairment are assumed to be transient [33] and not as strong as radiation-related impairments. Moreover, neurotoxicity of chemotherapy is not always proved in studies investigating this problem [34], and subjective complaints of patient seems to be more related to emotional distress and fatigue than to formal dysfunctions [35].

One other important goal of our project is to improve the detection of cognitive defects at early stage. Current formal assessment of cognitive status lasts between 1 and 2 h or more, and cannot be performed as often as needed in this population because of an important learning-effect. The *Compurterised Speed Cognitive Test (CSCT)* is a quick cognitive assessment tool validated in a population of patients presenting multiple sclerosis. The CSCT just need few minutes to be performed and appears to be a potential useful tool in the patient follow-up [36]. This test use in the neuro-oncologic context could importantly improve the detection of cognitive impairments and decrement. Our study will help to validate its use in our population by comparing the CSCT to a more complete battery at baseline time before radiotherapy and during the follow-up after treatment. Several biomarkers of neurotoxicity will be also tested in our population in order to try to individualize prognostic biomarkers of leukoencephalopathy.

Prospective methodology of data collection and the choice of a homogenous population will allow us to analyze precisely the selected outcomes, avoiding potential bias linked to retrospective studies. However, one limit of our study is to focus only on patients with high grade of glioblastoma, who are known to have a very short life expectancy, with a median expected survival being less than 18 months [37]. Nevertheless, it seems that leukoencephalopathy could appear as soon as within the 6 first months after radiotherapy. Furthermore, the rather large number of included patients will allow us to characterize cognitive defects at short and medium term.

With a better screening and understanding of neurotoxicity, the treatment regiments could be adapted to risk factors present for each patient in order to reduce complication. More, preventive actions and cares could be developed to reduce the burden of cognitive deficits and preserve the quality-of-life and autonomy for patient at risk.

## Conclusion

Leukoencephalopathy is one of the most frequent mid- to long-term complications of cranial radiotherapy. However, its incidence is not well established and the pathophysiology remains poorly understood. By an original multidisciplinary approach, the study EpiBrainRad aims to improve knowledge about this condition to facilitate the detection and prevention of radio-induced impairments. The main challenge is to preserve quality-of-life after cancer treatments, which involves to study risk factors and development of such complications.

## Abbreviations

CSCT: Computerised Speed Cognitive Test.
MRI: Magnetic Resonance Imagery
RT: Radiotherapy
